# The valuation of currency options by fractional Brownian motion

**DOI:** 10.1186/s40064-016-2784-2

**Published:** 2016-07-21

**Authors:** Foad Shokrollahi, Adem Kılıçman

**Affiliations:** Department of Mathematics and Statistics, University of Vaasa, 65101 Vaasa, Finland; Department of Mathematics, University Putra Malaysia (UPM), 43300 Serdang, Selangor Malaysia

**Keywords:** Black–Scholes model, Fractional Brownian motion, Currency option, Option pricing

## Abstract

This research aims to investigate a model for pricing of currency options in which value governed by the fractional Brownian motion model (*FBM*). The fractional partial differential equation and some Greeks are also obtained. In addition, some properties of our pricing formula and simulation studies are presented, which demonstrate that the *FBM* model is easy to use.

## Background

A currency options refers to an agreement that gives right to the holder in order to buy or sell a defined amount of foreign currency at a constant exercise price on option exercise. American options are traded at any time before they expire. European options can be exercised only during a specified period immediately before expiration.

Black and Scholes (1973) put forward option pricing in 1973, which leads to be studied by different scholars (Dravid et al. 1993; Toft and Reiner 1997; Kwok 2000; Duan and Wei 1999) claim that two issues in stock markets are not able to be presented clearly in this option pricing introduced by *BS* in accordance with Brownian motion (*BM*). These concepts refer to asymmetric leptokurtic features and the volatility smile. In view of this, the *BS* model was improved by Garman and Kohlhagen (1983) in order to assess European currency options by considering two prominent features;

1. The market volatility estimation of an underlying as obvious as price and time functioning void of referring to the characteristics of a particular investor directly. These characteristics could be functions of utility, measures of risk aversion, or yield expecting.
2. Strategy of self-replicating or hedging.

However, it is significant to note that the mispriced currency options by the *G*–*K* model were also substantiated in some studies (Cookson 1992). The most important reason of inappropriateness of this model for stock markets is the fact that the currencies are different from stocks so that the currency behavior is not captured by geometric Brownian motion (Ekvall et al. 1997). To tackle this problem regarding pricing currency options, various models were recommended by modifying the *G*–*K* model (Rosenberg 1998; Sarwar and Krehbiel 2000; Bollen and Rasiel 2003; Shokrollahi and Kılıçman 2014a, b, 2015).

In view of this, the independency of logarithmic returns of the exchange rate was pointed out in all these studies along with the distribution of normal random variables. In addition, the empirical studies reveal that the logarithmic returns disseminations in the asset markets widely manifest excess kurtosis with high possibility of mass around the origin and in the tails, and indicate low possibility in the flanks in comparison with normal distribution of data. It means that financial return series include the properties, which are not normal, independent, linear and are self-similar, with heavy tails. Both autocorrelations and cross-correlations and also volatility clustering are considered to these properties.

In this regard, two fundamental features are considered in *FBM* namely self-similarity and long-range dependence. Then, employing this process is more feasible in terms of capturing the behavior from financial asset (Carbone et al. 2004; Wang et al. 2010). Although, *FBM* is neither a semi-martingale nor a Markov process then, we are not able to employ the conventional stochastic calculus for analyzing it. Fortunately, the research interest in this field was re-encouraged by new insights in stochastic analysis based on the Wick integration (see Hu and Øksendal 2003) called the fractional-Ito-integral. Using this type of stochastic integration (Hu and Øksendal 2003) proofed that the fractional Black–Scholes market presents no arbitrage opportunity and is complete. However, Björk and Hult (2005) argued that the use of *FBM* in this context does not make much economic sense because, while Wick integration leads to no arbitrage, the definition of the corresponding self-financing trading strategies is quite restrictive and, for example, in the setup of Elliott and Van der Hoek (2003), the simple buy-and-hold strategy is not self-financing. We noted that this arbitrage example in discrete-time does not, however, rule out the use of *FBM* in finance. For example, Bender et al. (2007) showed that the existence of arbitrage opportunities depends very much on the definition of the admissible trading strategies. Furthermore, Bender et al. (2008) stated that the financial market does not admit arbitrage opportunities in a class of trading strategies if a continuous price process has the conditional small ball property and pathwise quadratic variation. Hence it is not too hard to accept this idea: some restrictions are sufficient to exclude arbitrage in the fractional Brownian market. Indeed, some authors have used the geometric *FBM* to capture the behavior of underlying asset and to obtain fractional Black–Scholes formulas for pricing options, including Necula (2002) and Bayraktar et al. (2004).

In this paper, the pricing formula is investigated for pricing currency options by using the *FBM* model. Furthermore, we obtain risk neutral valuation model and fractional Black–Scholes equation. Some properties and numerical studies of our pricing formula are also analyzed. “Preparations” section deals with the definition and features of the *FBM* process, and some results regarding quasi-conditional expectation are also investigated. In “Pricing model” section, option pricing formula for the European currency options is derived by the *FBM* model. “Properties of pricing formula” section describe the fractional differential equation and also investigates some Greeks of our model. We show empirical studies and simulation in “Numerical studies” section in order to indicate the efficiency of the *FBM* model and final section of the paper is “Conclusion”.

## Preparations

This section deals with some assumptions and definitions which is needed for this study. For get more information you can see Necula (2002), Cheridito (2003), Mishura (2008), and Hu and Øksendal (2003).

### **Definition 1**

A *FBM*, $B_H(t)$ with Hurst exponential $H\in (0,1)$ under the probability space $(\Omega ,F,P)$ is a continuous Gaussian process with these features:

1. $$B_H(0)=0$$.
2. $$E[B_H(t)]=0$$ for all $$t\ge 0$$.
3. $$cov[B_H(t)B_H(s)]=\frac{1}{2}\left[ t^{2H}+s^{2H}-|t-s|^{2H}\right] $$ for all $$s,t\ge 0$$.
4. If $H=\frac{1}{2}$ the $B_H(t)$ is equivalent to the Brownian motion.

Moreover, $$E(B_H(t)-B_H(s))^2=|t-s|^{2H}$$ and $$B_H(t)$$ is stationary increments and is *H*-self-similar in the sense that $$B_H(ct) $$ and $$c^HB_H(t)$$ have the similar distribution for every $$c>0$$. If $$H>\frac{1}{2}$$ the process $$B_H(t)$$ represents long-range correlation, by the following definition:

1 $$\begin{aligned} \sum _{m=1}^\infty E\left[ B_H(1)(B_H(m+1)-B_H(m))\right] =\infty . \end{aligned}$$

Now, suppose $(\Omega ,F,P)$ be a probability field such that $B_t^H$ is a *FBM* with respect to *P*, Some results represented that is required for the following (see Necula 2002).

### **Lemma 2**

*Consider the fractional differential equation*

2 $$\begin{aligned} dS_t=\mu S_tdt+\sigma S_t dB_t^H\qquad S_0=S, \end{aligned}$$

*then*

3 $$\begin{aligned} S_t=S_0\exp \left( \mu t+\sigma B_t^H-\frac{1}{2}\sigma ^2t^{2H}\right) . \end{aligned}$$

### **Lemma 3**

*Let*$0<t<T$*and*$\sigma \in \ {\mathbb {C}}$*then*

4 $$\begin{aligned} \widetilde{E}_t\left[ e^{\sigma B_T^H}\right] =e^{\sigma B_T^H+\frac{\sigma ^2}{2}\left( T^{2H}-t^{2H}\right) }, \end{aligned}$$

*where*$\widetilde{E}_t$*shows the quasi-conditional expectation under risk-neutral measure.*

### **Lemma 4**

*Supposefbe a function such that*$\widetilde{E}_t\left[ f(B_T^H)\right] <\infty$*. Thus for each*$0<t\le T$*and*$\sigma \in \ {\mathbb {C}}$*, we have*

5 $$\widetilde{E}_t\left[ f\left( \sigma B_T^H\right) \right]= \int _R\frac{1}{\sqrt{2\pi \sigma ^2\left( T^{2H}-t^{2H}\right) }} \times \exp \left[ -\frac{\left( x-\sigma B_t^H\right) ^2}{2\sigma ^2\left( T^{2H}-t^{2H}\right) }\right] f(x)dx.$$

*Let*$f(x)=\mathbf 1 _A$*thus, the following corollary is obtained.*

### **Corollary 5**

*Assume*$$A\in B(R)$$*. Therefore*

6 $$\widetilde{E}_t\left[ \mathbf 1 _A\left( \sigma B_T^H\right) \right]= \int _R\frac{1}{\sqrt{2\pi \sigma ^2\left( T^{2H}-t^{2H}\right) }} \times \exp \left[ -\frac{\left( x-\sigma B_t^H\right) ^2}{2\sigma ^2\left( T^{2H}-t^{2H}\right) }\right] \mathbf 1 _A(x)dx.$$

*Assume*$\theta ,w\in R$*. Then, this process considered*

7 $$\begin{aligned} Z_t^*=\theta \left( B_t^H\right) ^*=\theta B_t^H+\theta ^{2H}, \qquad 0\le t\le T. \end{aligned}$$

*According to the Girsanov formula, there is a measure*$P^*$*such that*$Z_t^*$*is a newFBM. We will denote*$E_t^*[.]$*is a quasi-conditional expectation under*$P^*$*. Consider*

8 $$\begin{aligned} X_t=exp\left( -\theta B^H_t-\frac{\theta ^2}{2}t^{2H}\right) . \end{aligned}$$

### **Lemma 6**

*Letfbe a function such that*$\widetilde{E}_t[f(\theta B_t^H)]\le \infty$*. Thus for each*$t\le T,$

9 $$\begin{aligned} \widetilde{E}_t^* \left[ f\left( \theta B_T^H\right) \right] = \frac{1}{X_t}\widetilde{E}_t \left[ f \left( \theta B_T^H \right) X_T\right] . \end{aligned}$$

### **Theorem 7**

*The price at every time*$t\in [0,T]$*of a bounded*$F_T^H$*-measurable claim*$F\in L^2$*as follows*

10 $$\begin{aligned} F_t=e^{-r(T-t)}\widetilde{E}_t[F], \end{aligned}$$

*wherershows the fixed rate of riskless interest.*

## Pricing model

Since, the system in finance is considered as an intricate system in investments in which investors avoid to make instant decisions after obtaining financial information in a fractional system. It means that achieving information to its threshold limit value is the major criteria for making decisions of investors rather than financial information with high flexibility. The asymmetric leptokurtic and long memory properties result from this behavior. In this regard, the beneficial model seems to be *FBM* model.

To derive the new currency option pricing formula in a fractional market. The following hypothesis will be provided:

1. there are no transaction costs or taxes;
2. security trading is continuous;
3. The rate of domestic interest $r_d$ and the rate of foreign interest $r_f$ are known and fixed throughout time;
4. There are no riskfree arbitrage opportunities.

Now, we consider a fractional Black–Scholes currency market that has two investments:

- a money market account
  11 $$\begin{aligned} dM_t=r_dM_tdt, \end{aligned}$$
  where $r_d$ show the rate of domestic interest.
- a stock whose price satisfies the following equation:
  12 $$\begin{aligned} dS_t=\mu S_t+\sigma S_td\widehat{B}_t^H \qquad 0<t\le T\qquad S_0=S>0, \end{aligned}$$
  where $\frac{1}{2}<H<1$ is Hurst parameter.

Let $B_t^H=\frac{\mu +r_f-r_d}{\sigma }t+\widehat{B}_t^H$, hence respect to risk-neutral measure we have:

13 $$\begin{aligned} dS_t=\left( r_d-r_f \right) S_t+\sigma S_td{B}_t^H \qquad 0<t\le T\qquad S_0=S>0. \end{aligned}$$

Then, the solution for Eq. (13) is

14 $$\begin{aligned} S_t=S_0\exp \left( \left( r_d-r_f \right) t+\sigma B_t^H-\frac{1}{2}\sigma ^2t^{2H}\right) . \end{aligned}$$

### **Theorem 8**

*The value at every*$t\in [0,T]$*of a European call currency option with exercise priceKand expirationTis given by*

15 $$\begin{aligned} C(t,S_t)=S_t e^{-r_f(T-t)}\Phi (d_1)-Ke^{-r_d(T-t)}\Phi (d_2), \end{aligned}$$

*where*

16 $$\begin{aligned} d_1\,&=  \,\frac{\ln \left( \frac{S_t}{K}\right) + \left( r_d-r_f\right) (T-t) + \frac{\sigma ^2}{2}\left( T^{2H}-t^{2H}\right) }{\sigma \sqrt{T^{2H}-t^{2H}}} \nonumber \\ d_2\,&=\,\sigma \sqrt{T^{2H}-t^{2H}}. \end{aligned}$$

### **Corollary 9**

*The value of European put currency option is given by*

17 $$\begin{aligned} P(t,S_t)=Ke^{-r_d(T-t)}\Phi (-d_2)-S_t e^{-r_f(T-t)}\Phi (-d_1), \end{aligned}$$

*where*

18 $$\begin{aligned} d_1 &=  \frac{\ln \left( \frac{S_t}{K}\right) + \left( r_d-r_f\right) (T-t) + \frac{\sigma ^2}{2}\left( T^{2H}-t^{2H}\right) }{\sigma \sqrt{T^{2H}-t^{2H}}} \nonumber \\ d_2 &=  \sigma \sqrt{T^{2H}-t^{2H}}. \end{aligned}$$

## Properties of pricing formula

Assume that *V* is the value of currency options which depends just on *t* and $S_t$. Thus, the value of whole portfolio satisfies in the partial differential equation that present in this theorem.

### **Theorem 10**

*The value of a currency options*$V(t,S_t)$*satisfies in the followingPDE*

19 $$\begin{aligned} \frac{\partial V}{\partial t}+H\sigma ^2t^{2H-1}S_t^2\frac{\partial ^2 V}{\partial S_t^2}+\left( r_d-r_f \right) S_t\frac{\partial V}{\partial S_t}-r_dV=0. \end{aligned}$$

*Now, we discuss the properties of theFBMmodel such as Greeks, which summarize how option prices change with respect to underlying variables that are critically important in asset pricing and risk management. In addition, it can be used to rebalance the portfolio to achieve desired exposure to a certain risk. It is significant to note that, knowing the Greek, a particular exposure can be hedged from adverse changes in the market by employing the appropriate amount of other related financial instruments. Contrary to option prices, observed in the market, Greeks can not be found and have to be calculated by a model assumption. Typically, the Greeks are computed using a partial differentiation of the price formula* Shokrollahi et al. (2015, 2016).

### **Theorem 11**

*The Greeks can be written as*

20 $$\Delta =  \frac{\partial C}{\partial S_t}=e^{-r_f(T-t)}\Phi (d_1),$$

21 $$\nabla=  \frac{\partial C}{\partial K}=-e^{-r_d(T-t)}\Phi (d_2),$$

22 $$\rho _{r_d}=  \frac{\partial C}{\partial r_d}=K(T-t)e^{-r_d(T-t)}\Phi (d_2),$$

23 $$\rho _{r_f}=  \frac{\partial C}{\partial r_f}=S_t(T-t)e^{-r_f(T-t)}\Phi (d_1),$$

24 $$\begin{aligned} \Theta\,=\,  \frac{\partial C}{\partial t}=S_tr_fe^{-r_f(T-t)}\Phi (d_1)-Kr_de^{-r_d(T-t)}\Phi (d_2)\nonumber \\& \quad - S_te^{-r_f(T-t)}\frac{\sigma Ht^{2H-1}}{\sqrt{T^{2H}-t^{2H}}}\Phi '(d_1), \end{aligned}$$

25 $$\Gamma=  \frac{\partial ^2 C}{\partial S_t^2} = e^{-r_f(T-t)}\frac{\Phi '(d_1)}{S_t\sqrt{\sigma ^2 \left( T^{2H}-t^{2H}\right) }},$$

26 $$\vartheta _\sigma=  \frac{\partial C}{\partial \sigma }=S_te^{-r_f(T-t)}\sqrt{T^{2H}-t^{2H}}\Phi '(d_1).$$

*The Hurst parameterHplay a significant role in theFBMmodel. Then, we represents the influence of this parameter in the following theorm.*

### **Theorem 12**

*The impact of the Hurst parameter as follows*

27 $$\begin{aligned} \frac{\partial C}{\partial H}=S_te^{-r_f(T-t)}\frac{\sigma \left( T^{2H}\ln T-t^{2H}\ln t\right) }{\sqrt{T^{2H}-t^{2H}}}\Phi '(d_1). \end{aligned}$$

**Fig. 1:**
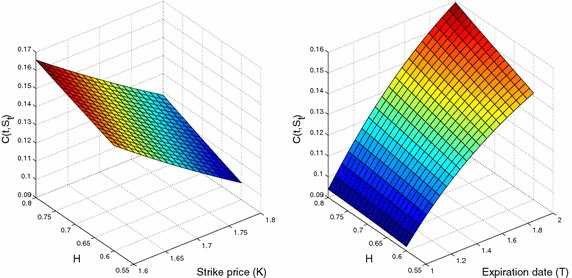
European Call currency option. Parameters fixed are $r_d=0.321,r_f=0.252,\sigma =0.21,T=2,k=0.1,K=1.625,S_t=1.512$, and $t=0.1$

*Fig.* 1*shows the impact of parameters on our pricing formula.*

*The following theorem presents the estimation of volatility byR* / *Smethod.*

### **Theorem 13**

*Assume*$0\le T_1<T_2$*be given, and let a partition of this interval is chosen,*$T_1=t_0<t_1< \cdots <t_n=T_2$*. Suppose*$S_{t_i}$*show the time series of observed price. Thus, the volatility of interval*$[T_1, T_2]$*is*

28 $$\begin{aligned} \sigma ^2=\frac{1}{T_2^H-T_1^H}\sum _{j=0}^{n-1} \left( \log \frac{S_{t_{j+1}}}{S_{t_{j}}}\right) ^2. \end{aligned}$$

### Remark 14

The relationship of call-put parity is given by

29 $$\begin{aligned} C(t,S_t)-P(t,S_t)=S_te^{-r_f(T-t)}-Ke^{-r_d(T-t)}. \end{aligned}$$

### Remark 15

The relationship of put-call parity satisfies

30 $$\begin{aligned} \frac{\partial C(t,S_t)}{\partial S_t}-\frac{\partial P(t,S_t)}{\partial S_t}=e^{-r_f(T-t)}. \end{aligned}$$

### Remark 16

The delta of spot exercise price has a space-homogeneity feature, such that for every $b>0$,

31 $$\begin{aligned} bC(t,S_t)=bS_t e^{-r_f(T-t)}\Phi (d_1)-bKe^{-r_d(T-t)}\Phi (d_2), \end{aligned}$$

and

32 $$\begin{aligned} bP(t,S_t)=bKe^{-r_d(T-t)}\Phi (-d_2)-bS_t e^{-r_f(T-t)}\Phi (-d_1). \end{aligned}$$

Furthermore, differenting both sides with under *b* and thus by $b=1$ we have

33 $$\begin{aligned} C(t,S_t)=S_t \frac{\partial C(t,S_t) }{\partial S_t}+K\frac{\partial C(t,S_t) }{\partial K}, \end{aligned}$$

and

34 $$\begin{aligned} P(t,S_t)=S_t \frac{\partial P(t,S_t) }{\partial S_t}+K\frac{\partial P(t,S_t) }{\partial K}. \end{aligned}$$

In fact, these equation is other model of the pricing currency option, when the value of stock is measured in a various unit. Moreover, $C'_{S_t}(t,S_t)$, $C'_{K}(t,S_t)$, $P'_{S_t}(t,S_t)$ and $P'_{K}(t,S_t)$ can be obtained by comparing this model with Eqs. (15), (17). These methods gives a new model for calculate delta.

## Numerical studies

This section deals with how implement the *FBM* model and shows the impact of Hurst parameter *H*. In the present study, we consider the real call currency options values from Philadelphia Stock exchange (*PHLX*) in order to investigate some information concerning our pricing formula. By applying the R/S method, we estimate the exponent parameter for EUR/USD and then we obtain $H=0.6102$. Furthermore, the volatility estimation is obtained by utilizing the historical volatility as follows;

35 $$ L_i=  \ln \left( \frac{q_{i+1}}{q_i}\right), $$

36 $$\sigma=  \sqrt{\frac{\sum (L_i-\overline{L})^2}{N-1}}, \qquad \overline{L}=\frac{1}{N}\sum L_i, $$

where $q_i$ show the daily value of exchange rate.

These data are extracted from 01/06/2010 to 01/12/2010 (six months) with the following parameters:

$$K=1.35, \sigma =0.1201, r_d=0.0231, r_f=0.0352, T=0.5$$, and $$ t=0.1$$. We use the MATLAB software for obtaining results by different models such as *G*–*K*, *BS* and *FBM* models. The values calculated by these models are represented in Table 1, where $$P_{Actual}$$ indicates the price of call currency options from *PHLX*, and the $$P_{BS}$$ is the values computed by the *BS* model. In addition, the $$P_{FBM}$$ points to the values calculated by *FBM* model. According to Table 1 our findings are more consistent with the actual price rather than the results of the other models. These properties reveal that our *FBM* model is able to get the behavior from financial market, which leads to creation of a satisfactory currency pricing model.

**Table 1:** Results by different pricing models

Exchange rate	$P_{BS}$	$P_{FBM}$	$P_{Actual}$
1.351	0.0377	0.0358	0.0338
1.357	0.0408	0.0388	0.0362
1.362	0.0433	0.0414	0.0391
1.368	0.0464	0.0444	0.0423
1.373	0.0490	0.0470	0.0456
1.379	0.0521	0.0501	0.0484
1.383	0.0542	0.0522	0.0503
1.389	0.0573	0.0553	0.0537
1.392	0.0589	0.0569	0.0548
1.398	0.0620	0.0601	0.0589

To further understand the preference of the *FBM* model, we calculated the theoretical prices of the our pricing formula and then we compare it with derived results from the *G*–*K* model and the *BS* model. For our propose, these parameter valuation are selected: $r_d=0.0210, r_f=0.0320,\sigma =0.1050, t=0.1, H=0.78 ,S_t=49$ for out-of-the-money case, $S_t=61$ for in-the-money case with different exercise price $K\in [50,60]$ and expiration date, $T\in [0.11,20]$.

**Fig. 2:**
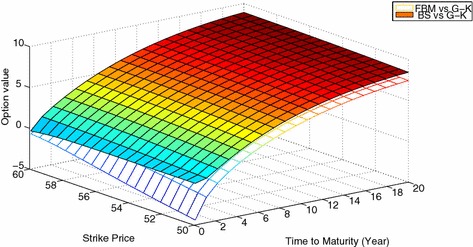
Relative difference among the *G*–*K* model, the *FBM* model and *BS* model in the in-the-money case

**Fig. 3:**
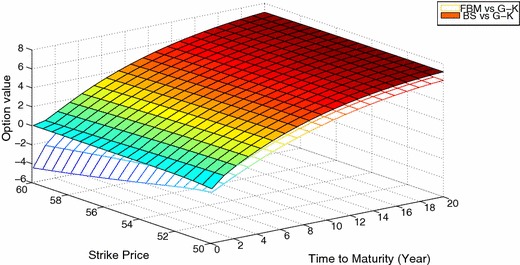
Relative difference among the *G*–*K* model, the *FBM* model and *BS* model in the out-of-the-money case

Figures 2 and 3 show the theoretical value discrepancy by the *G*–*K* model, *FBM* model and *BS* model, for in-the- money case and out-of-the-money case, respectively. These figures reveal that our pricing model are better matched with the *G*–*K* model. Then, from Table 1 and Figs. 2 and 3, we can conclude that our *FBM* model seems reasonable.

## Conclusion

This study provided a new framework for pricing currency options in accordance with the *FBM* model to capture long-memory property of the spot exchange rate. In addition, a obtained a new formula for pricing European call currency options and the volatility estimation were presented. Some certain features and Greeks of currency options model are also obtained. Finally, we reported the empirical results for several models, which demonstrate that the *FBM* model would be reasonable.

## Appendix

### Proof of Theorem 8

In a risk neutral world, from Theorem 7 a European call currency option with maturity *T* and strike price *K* can be display as

37 $$\begin{aligned} C(t,S_t) &=  \widetilde{E}_t \left[ e^{-r_d(T-t)}(S_T-K)^+\right] \nonumber \\ &=  e^{-r_d(T-t)}\widetilde{E}_t\left[ S_T\mathbf 1 _{S_T>K}\right] -Ke^{-r_d(T-t)}\widetilde{E}_t\left[ \mathbf 1 _{S_T>K}\right] . \end{aligned}$$

We will first consider $\widetilde{E}_t[\mathbf 1 _{S_T>K}]$. By setting

38 $$\begin{aligned} d_2^*=\ln \frac{K}{S}-\left( r_d-r_f \right) T+\frac{\sigma ^2}{2}T^{2H}. \end{aligned}$$

From Eq. (3), we have

39 $$\begin{aligned} S_t=S_0\exp \left( \mu t+\sigma B_t^H-\frac{1}{2}\sigma ^2t^{2H}\right) . \end{aligned}$$

Then

40 $$\begin{aligned} \widetilde{E}_t \left[ \mathbf 1 _{S_T>K}\right] &=  \widetilde{E}_t \left[ \mathbf 1 _{x>d_2^*} \left( \sigma B_t^H\right) \right] \nonumber \\ &=  \int _{d_2^*}^{+\infty }\frac{1}{\sqrt{2\pi \sigma ^2 \left( T^{2H}-t^{2H}\right) }} \exp \left[ -\frac{\left( x-\sigma B_t^H\right) ^2}{2\sigma ^2 \left( T^{2H}-t^{2H}\right) }\right] dx\nonumber \\ & =  \int _{\frac{d_2^*-\sigma B_t^H}{\sqrt{\sigma ^2 \left( T^{2H}-t^{2H} \right) }}}^{+\infty }\frac{1}{\sqrt{2\pi }} e^{-\frac{z^2}{2}}dz\nonumber \\ & =  \int _{-\infty }^{\frac{\sigma B_t^H-d_2^*}{\sqrt{\sigma ^2 \left( T^{2H}-t^{2H}\right) }}} \frac{1}{\sqrt{2\pi }}e^{-\frac{z^2}{2}}dz=\Phi (d_2). \end{aligned}$$

where $z^2=\frac{(x-\sigma B_t^H)^2}{\sigma ^2(T^{2H}-t^{2H})}$, thus $x>d_2^*$ means that $z>\frac{d_2^*-\sigma B_t^H}{\sqrt{\sigma ^2(T^{2H}-t^{2H})}}$ and the last equality follows since $\sigma B_t^H=\ln \frac{K}{S}-(r_d-r_f)t+\frac{\sigma ^2}{2}t^{2H}$.

Now, we consider $\widetilde{E}_t[S_T\mathbf 1 _{S_T>K}]$; setting

41 $$\begin{aligned} \sigma \left( B_t^H\right) ^*=\sigma \left( B_t^H-\sigma t^{2H}\right) . \end{aligned}$$

Let

42 $$\begin{aligned} X_t=S\exp \left( \sigma B_t^H-\frac{1}{2}\sigma ^2 t^{2H}\right) . \end{aligned}$$

Then we have $X_t=e^{-rt}S_t$. According to the Lemma 6, we obtain

43 $$\begin{aligned} \widetilde{E}_t \left[ S_T\mathbf 1 _{S_T>K}\right] &=  e^{rt}\widetilde{E}_t \left[ X_T\mathbf 1 _{x>d_2^*} \left( \sigma B_T^H \right) \right] \nonumber \\&=  e^{rt}X_t\widetilde{E}_t^* \left[ \mathbf 1 _{x>d_2^*} \left( \sigma B_T^H \right) \right] \nonumber \\&= e^{rt}X_t\widetilde{E}_t^* \left[ \mathbf 1 _{S_T>K}\right] . \end{aligned}$$

But

44 $$\begin{aligned} \ln S_T &=  \ln S+\left( r_d-r_f\right) T+\sigma B_T^H-\frac{1}{2} \sigma ^2T^{2H}\nonumber \\&=  \ln S+ \left( r_d-r_f\right) T+\sigma \left( B_T^H\right) ^*+\frac{1}{2}\sigma ^2T^{2H}. \end{aligned}$$

By setting $d_1^*=\ln \frac{K}{S}-(r_d-r_f)T-\frac{1}{2}\sigma ^2T^{2H}$, we obtain

45 $$\begin{aligned} \widetilde{E}_t^* \left[ \mathbf 1 _{S_T>K}\right] &= \widetilde{E}_t \left[ \mathbf 1 _{x>d_1^*}\left( \sigma \left( B_t^H \right) \right) ^*\right] \nonumber \\&= \int _{d_1^*}^{+\infty }\frac{1}{\sqrt{2\pi \sigma ^2 \left( T^{2H}-t^{2H}\right) }}\exp \left[ -\frac{\left( x-\sigma (B_t^H))^*\right) ^2}{2\sigma ^2 \left( T^{2H}-t^{2H}\right) }\right] dx \nonumber \\ &= \int _{\frac{d_1^*-\sigma (B_t^H))^*}{\sqrt{\sigma ^2 \left( T^{2H}-t^{2H}\right) }}}^{+\infty }\frac{1}{\sqrt{2\pi }} e^{-\frac{z^2}{2}}dz\nonumber \\ &= \int _{-\infty }^{\frac{\sigma B_t^H-d_2^*}{\sqrt{\sigma ^2 \left( T^{2H}-t^{2H}\right) }}}\frac{1}{\sqrt{2\pi }} e^{-\frac{z^2}{2}}dz=\Phi (d_1). \end{aligned}$$

The last equality follows since

46 $$\begin{aligned} \sigma B_t^H &=  \ln \frac{S_t}{S}-\left( r_d-r_f \right) t+\frac{1}{2}\sigma ^2t^{2H}\nonumber \\ \sigma \left( B_t^H\right) ^* &= \sigma \left( B_t^H-\sigma t^{2H}\right) . \end{aligned}$$

Then

47 $$\begin{aligned} \widetilde{E}_t^* \left[ \mathbf 1 _{S_T>K}\right] = e^{\left( r_d-r_f \right) T}X_t\Phi (d_1)=S_te^{\left( r_d-r_f \right) (T-t)}\Phi (d_2). \end{aligned}$$

$$\square $$

### Proof of Theorem 10

Let $V(t,S_t)$ be the price of the currency derivatives at time *t* and let $\Pi$ be the portfolio value. Then we have

48 $$\begin{aligned} \Pi _t=V(S_t,t)-\Delta S_t. \end{aligned}$$

Since

49 $$\begin{aligned} S_t=S_0\exp \left[ \mu T+\sigma B_T^H-\frac{1}{2}\sigma ^2T^{2H}\right] . \end{aligned}$$

Then

50 $$\begin{aligned} D_uS_\tau &= S_\tau D_u\left( \mu \tau +\sigma B_\tau ^H-\frac{1}{2}\sigma ^2\tau ^{2H}\right) \nonumber \\ &=  S_\tau \left[ D_u\left( \sigma B_\tau ^H\right) \right] ,\nonumber \\ D_u^\phi &= S_\tau H\sigma \tau ^{2H-1}. \end{aligned}$$

Hence we have

51 $$\begin{aligned} d\Pi _t &= dV(t,S_t)-\Delta (dS_t+r_fS_tdt)\nonumber \\&= \left( \frac{\partial V}{\partial t}+H\sigma ^2t^{2H-1}S_t^2\frac{\partial ^2V}{\partial S_t^2}+\mu S_t\frac{\partial V}{\partial S_t}\right) dt\nonumber \\& \quad +\sigma S_t\frac{\partial V}{\partial S_t}dB_t^H-\Delta \left( \mu S_t dt+\sigma S_tdB_t^H+r_fS_tdt\right) \nonumber \\ &= \left( \frac{\partial V}{\partial t}+H\sigma ^2t^{2H-1}S_t^2\frac{\partial ^2V}{\partial S_t^2}+\mu S_t\frac{\partial V}{\partial S_t}-\Delta \mu S_t-\Delta r_fS_t\right) dt\nonumber \\& \quad+ \left( \sigma S_t\frac{\partial V}{\partial S_t}-\Delta \sigma S_t\right) dB_t^H. \end{aligned}$$

For eliminate the stochastic noise we choose $\Delta =\frac{\partial V}{\partial S_t}$, then

52 $$\begin{aligned} d\Pi _t=\left( \frac{\partial V}{\partial t}+H\sigma ^2t^{2H-1}S_t^2\frac{\partial ^2V}{\partial S_t^2}-\Delta r_fS_t\right) dt. \end{aligned}$$

The return of an amount $\Pi _t$ invested in bank account equal to $r_d\Pi _tdt$ at time *dt*. For absence of arbitrage these values must be same, thus

53 $$\begin{aligned} \left( \frac{\partial V}{\partial t}+H\sigma ^2t^{2H-1}S_t^2\frac{\partial ^2V}{\partial S_t^2}- r_fS_t\frac{\partial V}{\partial S_t}\right) dt=r_d\Pi _tdt. \end{aligned}$$

Since $$\Pi _t=V(t,S_t)-\Delta S_t$$, hence

54 $$\begin{aligned} \frac{\partial V}{\partial t}+H\sigma ^2t^{2H-1}S_t^2\frac{\partial ^2V}{\partial S_t^2}- r_fS_t\frac{\partial V}{\partial S_t}dt=r_d\left(V-S_t\frac{\partial V}{\partial S_t}\right), \end{aligned}$$

so

55 $$\begin{aligned} \frac{\partial V}{\partial t}+H\sigma ^2t^{2H-1}S_t^2\frac{\partial ^2V}{\partial S_t^2}+\left( r_d-r_f \right) S_t\frac{\partial V}{\partial S_t}-r_dV=0. \end{aligned}$$

$$\square $$

### Proof of Theorem 11

First, we derive a general formula. Let *y* be one of the influence factors. Thus we have

56 $$\begin{aligned} \frac{\partial C}{\partial y} &= \frac{\partial S_te^{-(r_f)(T-t)}}{\partial y}\Phi (d_1)+S_te^{-r_f(T-t)}\frac{\partial \Phi (d_1)}{\partial y}\nonumber \\& \quad - \frac{\partial Ke^{-r_d(T-t)}}{\partial y}\Phi (d_2)-Ke^{-r_d(T-t)}\frac{\partial \Phi (d_2)}{\partial y}. \end{aligned}$$

But

57 $$\begin{aligned} \frac{\partial \Phi (d_2)}{\partial y} & = \Phi '(d_2)\frac{\partial d_2}{\partial y}\nonumber \\ &= \frac{1}{\sqrt{2\pi }}e^{-\frac{d_2^2}{2}}\frac{\partial d_2}{\partial y}\nonumber \\ &= \frac{1}{\sqrt{2\pi }}\exp \left( -\frac{ \left( d_1-\sqrt{\sigma ^2 \left( T^{2H}-t^{2H}\right) }\right) ^2}{2}\right) \frac{\partial d_2}{\partial y}\nonumber \\ &= \frac{1}{\sqrt{2\pi }}e^{-\frac{d_1^2}{2}}\exp \left( d_1\sqrt{\sigma ^2 \left( T^{2H}-t^{2H}\right) }\right) \exp \left( -\frac{\sigma ^2 \left( T^{2H}-t^{2H}\right) }{2}\right) \frac{\partial d_2}{\partial y}\nonumber \\&= \frac{1}{\sqrt{2\pi }}e^{-\frac{d_1^2}{2}}\exp \left( \ln \frac{S_t}{K}+\left( r_d-r_f \right) (T-t)\right) \frac{\partial d_2}{\partial y}\nonumber \\&= \frac{1}{\sqrt{2\pi }}e^{-\frac{d_1^2}{2}}\frac{S}{K} \exp \left( \left( r_d-r_f \right) (T-t)\right) \frac{\partial d_2}{\partial y}. \end{aligned}$$

Then we have that

58 $$\begin{aligned} \frac{\partial C}{\partial y} &= \frac{\partial S_te^{-(r_f)(T-t)}}{\partial y}\Phi (d_1)-\frac{\partial Ke^{-r_d(T-t)}}{\partial y}\Phi (d_2)\nonumber \\& \quad + S_te^{-r_f(T-t)}\Phi '(d_1) \frac{\partial \sqrt{\sigma ^2 \left( T^{2H}-t^{2H}\right) }}{\partial y}. \end{aligned}$$

Substituting in (58) we get the desired Greeks. $\square$

### Proof of Theorem 12

59 $$\begin{aligned} \eta &= \frac{\partial C}{\partial H} = S_te^{-r_f(T-t)}\Phi '(d_1) \frac{\partial \sqrt{\sigma ^2 \left( T^{2H}-t^{2H}\right) }}{\partial H}\nonumber \\& = S_te^{-r_f(T-t)}\Phi '(d_1)\frac{\sigma \left( T^{2H}-t^{2H}\right) }{\sqrt{T^{2H}-t^{2H}}}. \end{aligned}$$

$$\square $$

### Proof of Theorem 13

Since

60 $$\begin{aligned} S_t=S_0\exp \left( \mu t+\sigma \widehat{B}_t^H-\frac{1}{2}\sigma ^2 t^{2H}\right) . \end{aligned}$$

Then

61 $$\begin{aligned} \log \frac{S_{t_{j+1}}}{S_{t_j}}=\left( \mu \left( t_{j+1}-t_j\right) + \sigma \left( \widehat{B}_{t_{j+1}}^H-\widehat{B}_{t_j}^H\right) - \frac{1}{2}\sigma ^2 \left( t_{j+1}^2-t_j^2\right) \right) . \end{aligned}$$

Hence the sum of the squares of the long return is

62 $$\begin{aligned} \sum _{j=0}^{n-1}\left( \log \frac{S_{t_{j+1}}}{S_{t_j}}\right) ^2 = \left( \mu \left( t_{j+1}-t_j\right) + \sigma \left( \widehat{B}_{t_{j+1}}^H - \widehat{B}_{t_j}^H\right) - \frac{1}{2}\sigma ^2 \left( t_{j+1}^2-t_j^2\right) \right) ^2. \end{aligned}$$

When the maximum step size $||\Pi ||=\max _{j=0,...,n-1}(t_j-t_{j-1})$ is small. The right side of (27) is approximately equal to $\sigma ^2(T_2^{H}-T_1^{H})$ and then

63 $$\begin{aligned} \sigma ^2\approx \frac{1}{T_2^{H}-T_1^{H}}\sum _{j=0}^{n-1}\left( \log \frac{S_{t_{j+1}}}{S_{t_j}}\right) ^2. \end{aligned}$$

$$\square $$
